# Tubular injury in diabetic kidney disease: a focus on regulated cell death

**DOI:** 10.3389/fendo.2026.1877238

**Published:** 2026-07-31

**Authors:** Hongwei Sun, Weina Chen, Huanwen Huang, Lei Chen, Shiyang He, Xing Wang, Shiying Xie

**Affiliations:** 1 Guangdong Provincial Key Laboratory of Tumor Interventional Diagnosis and Treatment, Zhuhai Institute of Translational Medicine, Zhuhai People’s Hospital (The Affiliated Hospital of Beijing Institute of Technology, Zhuhai Clinical Medical College of Jinan University), School of Bioengineering, Zunyi Medical University, Zhuhai, China; 2 Department of Pharmacy, The Fifth Affiliated Hospital (Zhuhai) of Zunyi Medical University, Zhuhai, Guangdong, China; 3 Department of Urology, 900th Affiliated Hospital of People's Liberation Army of China (PLA), Fuzhou, Fujian, China; 4 Department of Endocrinology and Metabolism, The Fifth Affiliated Hospital (Zhuhai) of Zunyi Medical University, Zhuhai, Guangdong, China; 5 Department of Nephrology and Rheumatology, The Fifth Affiliated Hospital (Zhuhai) of Zunyi Medical University, Zhuhai, Guangdong, China

**Keywords:** diabetic kidney disease, regulated cell death, tubular epithelial cell, tubular injury, crosstalk

## Abstract

Diabetic kidney disease (DKD), with the high prevalence of diabetes mellitus, has become the primary cause of end-stage renal disease. Tubular injury is one of the earliest characteristic changes and serves as a significant driving factor of renal function loss in DKD. Under physiological conditions, regulated cell death (RCD) preserves renal homeostasis by eliminating damaged tubular epithelial cells. Emerging evidence has demonstrated that abnormal RCD of tubular epithelial cells aggravates the development of DKD. Our review intends to elucidate recent discoveries regarding the pathogenetic role of the most studied RCD, such as apoptosis, necroptosis, pyroptosis, ferroptosis and autophagy, in DKD, and provide a theoretical foundation for future investigations as well as targeted drug design.

## 1 Introduction

In clinical practice, diabetic kidney disease (DKD) is diagnosed based on a persistent reduction in the estimated glomerular filtration rate (eGFR < 60 mL/min/1.73 m²) and/or the presence of sustained albuminuria (urinary albumin-to-creatinine ratio [UACR] ≥ 30 mg/g) for at least three months in patients with a documented history of diabetes (1–4). Chronic hyperglycemia, elevated glycated hemoglobin (HbA1c) levels, and dyslipidemia, particularly characterized by increased triglycerides and low-density lipoprotein cholesterol, are independent risk factors that directly accelerate eGFR decline and progression to end-stage kidney disease (ESRD) (3, 5). Furthermore, recent longitudinal cohort evidence confirms that increased HbA1c variability is highly predictive of progressive renal staging and a higher risk of DKD development (6). Abnormal serum lipid concentrations, specifically elevated total cholesterol (TC) and triglycerides (TG), closely correlate with worsening UACR and a poorer long-term renal prognosis (7). About 30% of patients with type 1 diabetes mellitus patients and 40% of patients with type 2 diabetes mellitus may eventually develop DKD (1, 8). With the increasing prevalence of diabetes mellitus, DKD has become the leading cause of ESRD, posing heavy economic pressure on individuals and public health systems (8, 9). Therefore, gaining deeper insights into the molecular mechanisms underlying the development of DKD is an urgent issue to address for developing novel therapies and improving patient outcome.

Glomerulosclerosis, tubular atrophy, and interstitial fibrosis are the primary pathological features of DKD (10). Previous investigations have largely focused on how glomerulopathy contributes to DKD progression (10). However, accumulating evidence in recent years reveals that tubular injury plays a crucial role in the pathological progress of DKD and is highly correlated with the progressive loss of renal function (1, 11). Furthermore, tubular abnormalities may even precede glomerular pathology in DKD (11). Despite intensive investigation, pathological mechanisms initiating and mediating tubular injury and degeneration in DKD remain poorly understood. Therefore, understanding the processes that lead to tubular injury is essential for the management and prevention of DKD.

**Figure 1:**
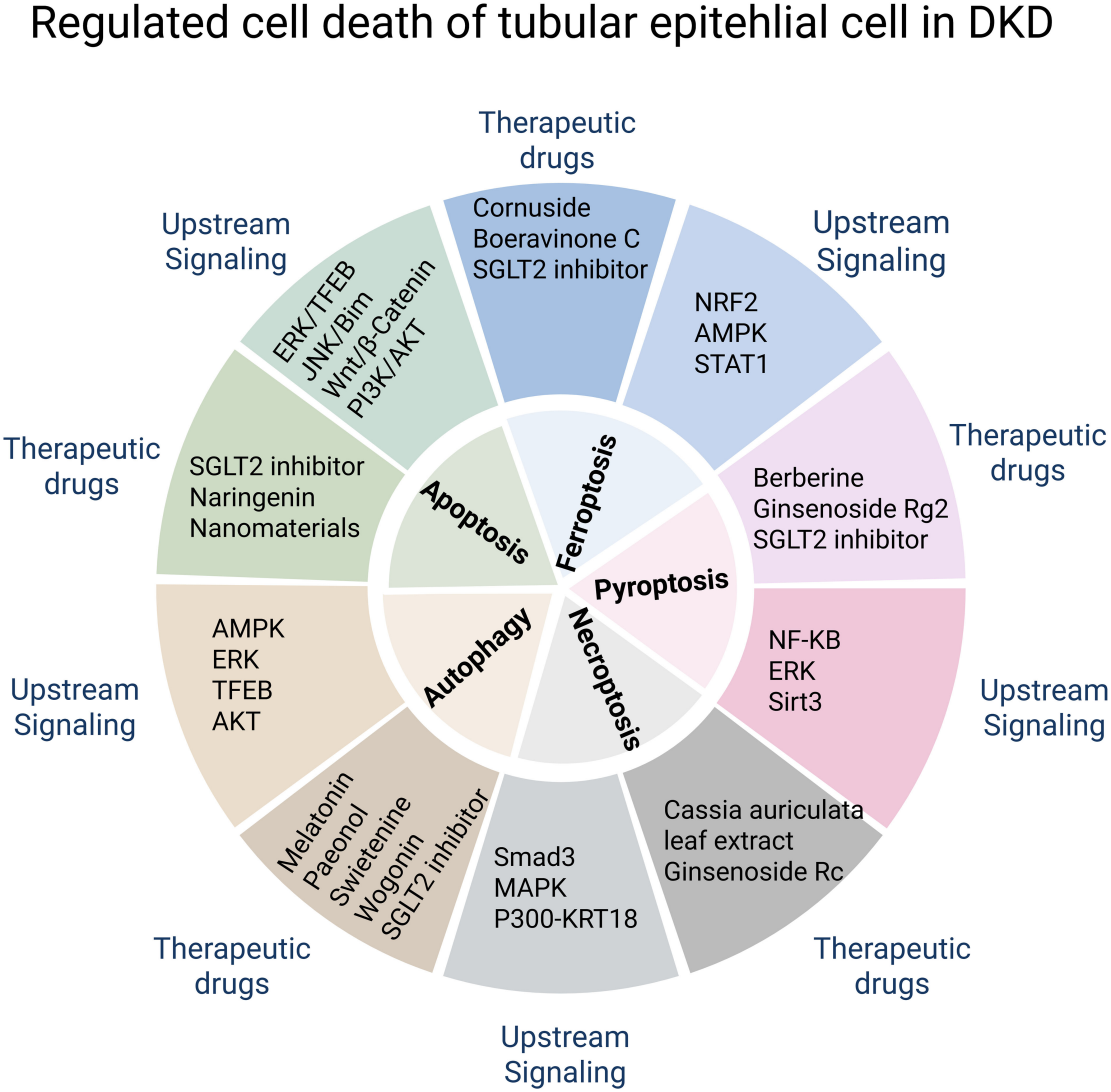
The upstream signaling and therapeutic drugs of RCD in TECs during DKD. The upstream signaling pathways regulating RCD and therapeutic drugs, such as SGLT2 inhibitor, GLP-1 receptor agonist, and natural products, targeting RCD in DKD are summarized. DKD, Diabetic Kidney Disease; SGLT2, Sodium-Glucose Cotransporter 2; NRF2, Nuclear Factor Erythroid 2-related Factor 2; AMPK, AMP-activated Protein Kinase; STAT1, Signal Transducer and Activator of Transcription 1; NLRP3, NLR Family Pyrin Domain Containing 3; NF-KB, Nuclear Factor Kappa B; ERK, Extracellular Signal-regulated Kinase; Sirt3, Sirtuin 3; Smad3, SMAD Family Member 3; MAPK, Mitogen-Activated Protein Kinase; P300-KRT18, p300-Keratin 18 complex; AKT, Protein Kinase B; TFEB, Transcription Factor EB; JNK, c-Jun N-terminal Kinase; Bim, Bcl-2-interacting mediator of cell death; Wnt, Wingless-related integration site; PI3K, Phosphoinositide 3-kinase. Created in BioRender.com.

**Figure 2:**
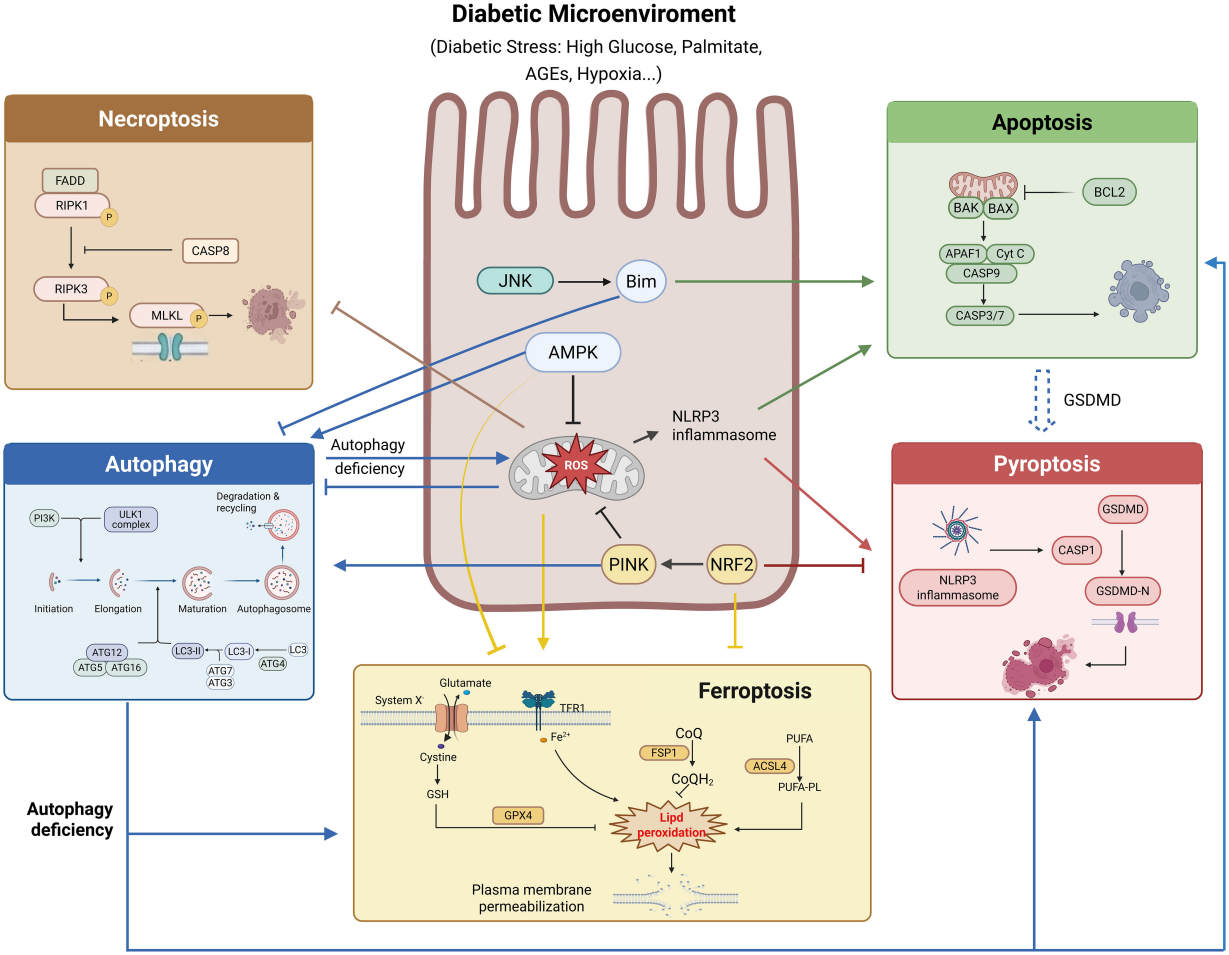
Crosstalk among RCD in TECs during DKD. Apoptosis, pyroptosis, ferroptosis, autophagy and necroptosis form an intricate regulatory network. Autophagy deficiency increases cellular susceptibility to apoptosis, pyroptosis and ferroptosis. GSDMD is a definitive molecular switch mediates the transition from apoptosis to pyroptosis, connecting non-inflammatory and inflammatory cell death. Autophagy deficiency elevates mitochondrial reactive oxygen species (mtROS) levels and sensitizes cells to apoptosis, pyroptosis and ferroptosis. Pyroptosis and ferroptosis act synergistically via shared signaling nodes, with mtROS serving as a pivotal common mediator. Pyroptosis and ferroptosis are regulated by NRF2, while PINK, the downstream regulator of NRF2, regulates autophagy. AMPK directly modulates autophagy, as well as regulates apoptosis, pyroptosis and ferroptosis via suppressing mtROS. Bim acts as a negative regulator of autophagy and a positive regulator of apoptosis. AGE, Advanced Glycation End-product; JNK, c-Jun N-terminal Kinase; Bim, Bcl-2-interacting mediator of cell death; AMPK, AMP-activated Protein Kinase; NLRP3, NLR Family Pyrin Domain Containing 3; PINK, PTEN-induced kinase; NRF2, Nuclear Factor Erythroid 2-related Factor 2; ROS, Reactive Oxygen Species; BAK, Bcl-2 Antagonist/Killer 1; BAX, Bcl-2-associated X protein; BCL2, B-cell Lymphoma 2; APAF1, Apoptotic Protease Activating Factor 1; Cyt C, Cytochrome C; CASP9, Caspase-9; CASP3, Caspase-3; CASP7, Caspase-7; CASP1, Caspase-1; GASDMD, Gasdermin D; GASDMD-N, Gasdermin D N-terminal domain; GSH, Glutathione; TFR1, Transferrin Receptor 1; GPX4, Glutathione Peroxidase 4; CoQ, Coenzyme Q; CoQH2, Ubiquinol (Reduced Coenzyme Q); ACSL4, Acyl-CoA Synthetase Long-Chain Family Member 4; PUFA, Polyunsaturated Fatty Acid; PUFA-PL, Polyunsaturated Fatty Acid-containing Phospholipid; ULK1, Unc-51 Like Autophagy Activating Kinase 1; PI3K, Phosphoinositide 3-kinase; ATG12, Autophagy-related protein 12; ATG5, Autophagy-related protein 5; ATG6, Autophagy-related protein 6 (Beclin-1); LC3-II, Microtubule-associated protein 1 light chain 3-II; LC3-I, Microtubule-associated protein 1 light chain 3-I; LC3, Microtubule-associated protein 1 Light Chain 3; ATG4, Autophagy-related protein 4; ATG3, Autophagy-related protein 3; ATG7, Autophagy-related protein 7; FADD, Fas-associated protein with Death Domain; RIPK1, Receptor-interacting Serine/Threonine-protein Kinase 1; RIPK3, Receptor-interacting Serine/Threonine-protein Kinase 3; CASP8, Caspase-8; MLKL, Mixed Lineage Kinase Domain-like protein. Created in BioRender.com.

Regulated cell death (RCD), known as the final stage of cells, occurs in response to specific stimuli or signals to maintain internal environmental stability (12, 13). Under physiological conditions, RCD preserves renal homeostasis by clearing aging, damaged, or malfunctioning cells (13). However, under a chronic glucolipotoxic environment, persistent hyperglycemia disrupts mitochondrial and endoplasmic reticulum homeostasis to trigger extensive oxidative stress and advanced glycation end-product accumulation, which directly activates various intracellular signaling cascades to initiate RCD pathways (14) (**Table 1**). Concurrently, the aberrant accumulation of ectopic lipids, such as palmitic acid, triggers local mitochondrial damage and lipid peroxidation to further accelerate these RCD processes (5). Emerging evidence has clarified that abnormal or imbalanced RCD of tubular epithelial cells (TECs) aggravates DKD by inducing functional and structural damage to renal tubule (33, 68–70). The dysregulation of these diverse RCD pathways is not only extensively documented in cellular and animal models (**Table 2**) but is also validated by transcriptomic and single-cell profiling of human clinical datasets. In this review, we summarize recent advancements in RCD research and provide a comprehensive overview of their contributions to the pathogenesis of DKD (**Figure 1**). Specifically, we summarized the distinct interconnected roles of pyroptosis, apoptosis, necroptosis, autophagy and ferroptosis related to tubular injury within DKD (**Figure 2**).

**Table 1:** Summary of regulated cell death (RCD) modalities and their key molecular regulators in DKD.

RCD	Canonical regulators	Positive regulators in DKD	Negative regulators in DKD
Apoptosis	CASP3, CASP7, CASP9, BAX, BAK, BCL2, APAF1, TP53, Cyt C	RTN1A (15), PGAM5 (16), CD36 (17), METTL14 (18), ATF5 (19), ClpP (20)	DsbA-L (21), miR-424-5p (22), miR-140-5p (23), APEH (24), UHRF1 (25), PKM2 (26)
Necroptosis	RIPK1, RIPK3, MLKL, FADD, CASP8, TNFα	ZBP1 (27), KRT18 (28, 29)	PINK1 (30), PPARα (31)
Autophagy	PI3K, LC3-II, p62, BECN1, ATG3, ATG4, ATG5, ATG7, ATG12, ATG16, ULK1, PINK, PARKIN	Klotho (32), PACS2 (33), VDR (34), SESN2 (35), ESRRA (36), GSTK1 (37)	TXNIP (38), ATF4 (39), P2Y2R (40), SMAD3 (41), XIAP (42), AARS1 (43)
Pyroptosis	GSDMD, NLRP3, IL1B, CASP1, PYCARD, IL18, GSDME	ALPK1 (44), IRE1α (45), PRR (46), ISG15 (47), SGK1 (48), HPGDS (49)	SIRT1 (50), PDIA4 (51), NRF2 (52), miR-342-3p (53), miR-23c (54), TFEB (55)
Ferroptosis	GPX4, FSP1, FTH1, ACSL4, TFR1, PTGS2, SLC7A11	AARS1 (56), ZIP14 (57), PRMT6 (58), STING (59), C/EBPα (60), CNPY2 (61)	NRF2 (62), AMPK (63), SIRT6 (64), VDR (65), USP9X (66), SIRT3 (67)

CASP3, Caspase 3; CASP9, Caspase 9; BAX, BCL2 Associated X; BAK, BCL2 Antagonist/Killer 1; BCL2, B-Cell Lymphoma 2; PARP1, Poly(ADP-Ribose) Polymerase 1; TP53, Tumor Protein P53; CYCS, Cytochrome C; RIPK1, Receptor Interacting Serine/Threonine Kinase 1; RIPK3, Receptor Interacting Serine/Threonine Kinase 3; MLKL, Mixed Lineage Kinase Domain Like Protein; FADD, Fas Associated Via Death Domain; CASP8, Caspase 8; TNFα, Tumor Necrosis Factor Alpha; LC3-II, Microtubule Associated Protein 1 Light Chain 3-II (Lipidated Form); p62, Sequestosome 1; BECN1, Beclin 1; ATG5, Autophagy Related 5; ATG7, Autophagy Related 7; ULK1, Unc-51 Like Autophagy Activating Kinase 1; PINK, PTEN Induced Kinase 1; PARKIN, Parkin RBR E3 Ubiquitin Protein Ligase; AMPK, AMP-Activated Protein Kinase; GSDMD, Gasdermin D; NLRP3, NLR Family Pyrin Domain Containing 3; IL1B, Interleukin 1 Beta; CASP1, Caspase 1; PYCARD, PYD And CARD Domain Containing; IL18, Interleukin 18; GSDME, Gasdermin E; GPX4, Glutathione Peroxidase 4; FSP1, Ferroptosis Suppressor Protein 1; FTH1, Ferritin Heavy Chain 1; ACSL4, Acyl-CoA Synthetase Long Chain Family Member 4; TFRC, Transferrin Receptor; PTGS2, Prostaglandin-Endoperoxide Synthase 2; SLC7A11, Solute Carrier Family 7 Member 11.

**Table 2:** Summary of diabetic kidney disease models and their characteristics and altered RCD pathways.

Model name/stimulus	Model class	Intervention	Characteristics of models	Altered RCD pathways
STZ diabetic mouse model	*In vivo*	Driven by multiple low-dose injections of streptozotocin to selectively destroy pancreatic β -cells	T1DM model; insulin deficiency; abrupt hyperglycemia, low body weight; moderate albuminuria; mesangial expansion, tubular injury, and renal fibrosis	Apoptosis (71–73), Necroptosis (74), autophagy (32, 39, 75), Pyroptosis (76–78), Ferroptosis (79–81)
db/db diabetic mouse model	*In vivo*	Driven by homozygous autosomal mutation in the leptin receptor gene	T2DM model; early-onset and severe obesity, insulin resistance, hyperlipidemia, high body weight and serum triglyceride, great tubular injury and progressive tubulointerstitial fibrosis;	Apoptosis (82, 83), Necroptosis (84), Autophagy (41, 85), Pyroptosis (46, 86, 87) Ferroptosis (79, 81)
eNOS^-/-^ db/db diabetic mouse model	*In vivo*	Driven by global endothelial nitric oxide knockout;	An accelerated nephropathy model; significant and early onset albuminuria, arteriolar hyalinosis, mesangiolysis, and focal segmental and nodular glomerulosclerosis, moderate hypertension; extensive tubular injury	Necroptosis (88)
High-glucose exposure	*In vitro*	30mM glucose exposure for 24–72 hours	Mimics clinical hyperglycemia; triggers cell viability loss, oxidative stress, mitochondrial ROS, ER stress, and cell hypertrophy; increases inflammation	Apoptosis (73, 89), Autophagy (90, 91), Necroptosis (29, 84), Ferroptosis (63, 92), Pyroptosis (44, 55)
Fatty acid exposure	*In vitro*	Palmitate acid-BSA (200 uM) for 24–48 hours	Mimics lipid overload/lipotoxicity; induces mitochondrial damage and lipid peroxidation; reduces cell viability; increases oxidative stress and mitochondrial ROS; increases inflammation	Apoptosis (93); Autophagy (94, 95); Pyroptosis (96); Necroptosis (84); Ferroptosis (95)
AGEs exposure	*In vitro*	AGE-BSA (100 μg/ml) for 12–24 hours	Mimics advanced glycation end-product accumulation; drives severe inflammation and cell viability loss; causes cell swelling and membrane rupture	Autophagy (41, 97, 98), Apoptosis (97, 99)

RCD, Regulated Cell Death; STZ, Streptozotocin; T1DM, Type 1 Diabetes Mellitus; T2DM, Type 2 Diabetes Mellitus; eNOS, Endothelial Nitric Oxide Synthase; ROS, Reactive Oxygen Species; ER, Endoplasmic Reticulum; BSA, Bovine Serum Albumin; AGE, Advanced Glycation End-product.

## 2 Apoptosis

### 2.1 Apoptotic signaling pathway

Apoptosis, an RCD type that has been most widely investigated and precisely regulated, is featured by cell shrinking, plasma membrane blebbing, chromatin condensation, and apoptotic bodies formation (12, 100). These apoptotic fragments are usually rapidly engulfed and degraded by surrounding macrophages to prevent inflammation (100).

In terms of mechanism, two key pathways are mainly responsible for activating apoptosis: the intrinsic pathway (mitochondria-mediated pathway) and the extrinsic pathway (death receptor-mediated pathway) (101, 102). The intrinsic apoptotic pathway responds to intracellular signals such as DNA damage or oxidative stress. These stimuli drive the accumulation of pro-apoptotic B-cell lymphoma 2 (Bcl-2) proteins on the outer mitochondrial membrane (OMM), thereby enhancing OMM permeabilization (102, 103). Subsequently, cytochrome c translocates from the mitochondrial intermembranous space into the cytoplasm, where they form the apoptosome and eventually trigger executioner caspases (104, 105). The extrinsic pathway is initiated by the binding of extracellular signals with transmembrane death receptors which directly recruits and activates caspases (101). The execution of apoptosis relies on the cascade activation of caspases, based on their function, these are grouped into initiator caspases (caspases-8, -9, -10) and executioner caspases (caspases-3, -6, -7) (105). Triggered by upstream signals, the initiator caspases undergo autocleavage, and the subsequently activated cleaved initiator caspases stimulate the downstream executioner caspases to execute apoptosis (106).

### 2.2 Apoptosis of tubular epithelial cell in DKD

Aberrant TEC apoptosis has been widely recognized as one of the core pathological events (1, 10). In DKD, cellular stress caused by abnormal stimulations, such as hyperglycemia, hyperlipidemia, and hypoxia, leads to mitochondrial dysfunction which eventually induces apoptosis. High-glucose (HG) incubation robustly induces apoptosis in human kidney-2 (HK-2) cells, as evidenced by a reduction in Bcl-2 expression, an upregulation of Bax, and an increased apoptotic rate (73). Exposure to saturated palmitate profoundly drives apoptosis in proximal tubular cells, as demonstrated by elevated cleaved caspase-3 and TUNEL-positive cells, both of which are significantly reversed by supplementing monounsaturated oleate (107). In streptozotocin (STZ)-treated mice and HG-exposed TECs, IGF2BP3 suppresses mitochondrial fission to inhibit TECs apoptosis by activating CAMK1 (71). HIF-1α overexpression or HO-1 agonist treatment suppresses TECs apoptosis by preventing hyperglycemia-induced mitochondrial membrane potential loss and mitochondrial fragmentation to eliminate tubular injury in DKD (108). In db/db mice and HG-exposed HK-2 cells, overexpression of mitofusin 2 restores mitochondrial function, suppresses mitochondrial oxidative phosphorylation, reduces tubular apoptosis and eventually alleviates diabetic kidney injury, and the reverse tendency is observed in mitofusin 2 short hairpin RNA treated db/db mice (82). Moreover, mitochondria-targeted antioxidant Mito-Tempo alleviates diabetic tubular injury and TECs apoptosis by reducing mitochondrial double-stranded RNA release and inhibiting the activation of the PKR/eIF2α signaling pathway (109), indicating that mitochondria quality control dysfunction is a significant upstreaming point in regulating apoptosis.

Beyond mitochondrial dysfunction, the association between apoptosis and endoplasmic reticulum (ER) stress has also been widely studied (37, 83, 110). In db/db mice and HG-treated TECs, ectonucleoside triphosphate diphosphohydrolase 5 overexpression inhibited apoptosis to preserve TECs via regulating the protein N-glycosylation rate in ER (83). The GSTK1/RETREG1 complex conferred protection against diabetic tubular damage and kidney injury via modulating ER stress and apoptosis through the induction of reticulophagy, thereby revealing that maintaining ER homeostasis is crucial for the control of apoptosis (37). Of note, oxidative stress serves as another crucial inductive factor of apoptosis (72, 111). DsbA-L interacts with catalase to reduce cellular oxidative stress, apoptosis, and renal tubular damage via improving peroxisomal function in HFD/STZ-induced diabetic mice (72). *In vitro*, overexpression of DsbA-L decreases the injury of HG and palmitic acid treated HK-2 cells by directly interacting with catalase (72). Moreover, nod-like receptor protein 3 (NLRP3) inflammasome activation is highly involved in apoptotic cell death (112, 113). Both *in vivo* and *in vitro* studies indicate that CD36 ameliorates HG induced TECs apoptosis via mediating NLRP3 inflammasome activation (112).

In terms of mechanism, multiple signaling pathways modulate apoptosis. JNK/Bim, Wnt/β-Catenin, PI3K/AKT and ERK/TFEB signaling pathways have been demonstrated to modulate TECs apoptosis in DKD (114–117). Metabolic disorder is also an upstreaming driver of apoptosis (118, 119). Sodium-glucose co-transporter 2 (SGLT2) inhibitor canagliflozin suppresses TECs apoptosis via maintaining metabolic homeostasis (119), while boeravinone C, an PPARα activator/agonist, can alleviate lipid accumulation and inflammation, thereby suppressing tubular apoptosis (120).

In recent years, novel regulatory factors of apoptosis, including non-coding RNA, estrogen receptor ER-α36, and various circulating proteins, have been identified (121–123), expanding our understanding of apoptosis regulatory network. For example, miR-200a-3p aggravates diabetic kidney injury through inducing tubular apoptosis via downregulating sirtuin 1 expression (121). By regulating PTEN and the PI3K/AKT axis, ER-α36 migrates the apoptotic death of tubular cells under HG conditions (122).

In the treatment of DKD, clinical therapeutic drugs have shown robust alleviation effect on tubular injury. SGLT2 inhibitors, such as canagliflozin empagliflozin and dapagliflozin, suppress TEC apoptosis to ameliorate diabetic kidney damage (119, 124, 125). Dapagliflozin treatment ameliorates HG-induced oxidative stress and apoptosis, as manifested by decreased ROS levels, fewer apoptotic nuclei, and downregulated Nox4 and TGF-β expression (124). The therapeutic effects of nanomaterials in treating DKD have been demonstrated. Zinc oxide nanoparticles ameliorate tubular injury by inhibiting apoptosis (126). Gold nanoparticles show a protective effect on DKD by targeting miR-192 and miR-21 to suppress TECs apoptosis (127). Traditional Chinese medicine and natural products have shown promising therapeutic effect on DKD. Agents such as quercetin, jujuboside A, cornuside, boeravinone C, cycloastragenol, and the JinChan YiShen TongLuo formula have been demonstrated to block TECs apoptosis through modulating mitochondrial function, ER stress, or key signaling pathways (117, 120, 128–131), exhibiting promising translational potential.

## 3 Necroptosis

### 3.1 Necroptotic signaling pathway

Necroptosis is a highly regulated, lytic form of RCD characterized by swelling of organelle and cellular, rupture of dying cells, and the subsequent release of intracellular contents and cytokines (132). Mechanistically, necroptosis can be triggered by a variety of extracellular and intracellular danger signals, including tumor necrosis factor-α (TNF-α), Fas ligand, interferons, or Toll-like receptor (TLR) ligands (133). Upon ligand binding, these stimuli initiate the assembly of a key multi-protein signaling complex composed of receptor-interacting protein kinase 3 (RIPK3) and receptor-interacting protein kinase 1 (RIPK1), called necrosome. Within the necrosome, RIPK1 interacts with and phosphorylates RIPK3, which subsequently recruits and phosphorylates mixed lineage kinase domain-like protein (MLKL). Upon activation, phosphorylated MLKL (p-MLKL) oligomerizes and translocates to the cell membrane, forming pores that disrupt electrochemical gradients, resulting in lytic cell rupture and the release of damage-associated molecular patterns (DAMPs), inflammatory cytokines and chemokines. Consequently, necroptosis creates a pro-inflammatory environment that compromises tissue integrity (134).

### 3.2 Necroptosis of tubular epithelial cell in DKD

Transcriptomic and single-nucleus RNA sequencing analyses have demonstrated significant enrichment and perturbations of the necroptosis pathway in diabetic kidney tissues, highlighting its core role in driving tubulointerstitial injury (27, 135). In rat proximal tubular cells (NRK-52E), HG conditions robustly activate the RIPK1/RIPK3/MLKL cascade, leading to severe cell death and inflammation (74). Treatment with Nec-1, a well-established necroptosis inhibitor, can inhibit cell death *in vitro*, and reduce kidney inflammation and attenuate renal dysfunction in HFD/STZ-induced diabetic mice (74). Apart from hyperglycemic, lipotoxicity is another prominent activator of tubular necroptosis (84). Exposure to fatty acids aggregates lipid accumulation and accelerates tubular cell necroptosis. The level of lipid deposition is closely correlated with necroptosis activation in the tubular compartment of patients with DKD (84). The RIPK1 inhibitor RIPA-56 significantly attenuates tubular epithelial cell necroptosis, reduces lipid deposition, and alleviates diabetic renal injury in STZ/HFD-treated db/db mice (84). In eNOS^−/−^ mice, RIPK3 blockade or RIPK3 deficiency has been shown to reduce kidney inflammatory cell infiltration and reduce myofibroblast activation and collagen deposition (88).

Upstream regulatory mechanisms of necroptosis in DKD involve distinct epigenetic, transcriptional, and microenvironmental factors. Epigenetic modifications, such as histone acetylation and lactylation, have emerged as vital regulators of tubular necroptosis. Under diabetic stress, histone acetyltransferase P300 transcriptionally activates Keratin 18 (KRT18) expression via promoting histone H3 lysine 18 acetylation (H3K18ac). Increased KRT18 then triggers the activation of the RIPK1/MLKL pathway to execute necroptosis. Knockdown or inhibition of P300 effectively can downregulate KRT18 expression and alleviate necroptosis (29). At the transcriptional level, the transcription factor ETS1 activates the expression of Z-DNA binding protein 1 (ZBP1). Upon induction, ZBP1 physically associates with RIPK3 to trigger necroptotic cell death; conversely, ZBP1 knockdown significantly attenuates tubular injury, inflammation, and fibrotic remodeling in *db/db* mice (27). The inflammatory microenvironment, heavily driven by macrophage polarization, is intimately linked to tubular necroptosis. Infiltrating M1 macrophages polarized via either Notch/NF-κB crosstalk or prostaglandin receptor EP3/Rho kinase signaling secrete pro-inflammatory cytokines that exacerbate tubular necroptosis, extracellular matrix deposition, and renal fibrosis (136, 137). Additionally, the SARS-CoV-2 nucleocapsid (N) protein physically binds Smad3 to promote its phosphorylation and nuclear translocation, thereby triggering the Smad3-RIPK3-MLKL pathway to induce severe tubular necroptosis and kidney injury in diabetic models (138).

Apart from canonical necroptosis inhibitors, several natural products have been shown to suppress necroptosis in DKD models. Concomitant administration of *Cassia auriculata* ethanolic leaf extract mitigates HG-induced TECs neroptosis and restores biochemical homeostasis, renal function, and structural integrity in diabetic rats (139). By competing for the lactate-binding site, Ginsenoside Rc, a bioactive constituent of *Panax ginseng*, suppresses the lactyltransferase activity of KRT18. Consequently, this intervention reduces the accumulation of histone lactylation and suppresses the expression of Fas, ultimately alleviating necroptotic cell death and renal impairment in DKD models (28).

## 4 Autophagy

### 4.1 Autophagic signaling pathway

Autophagy represents a preserved cellular mechanism by which cytoplasmic materials are engulfed within autophagosomes for lysosomal degradation and recycling (140). It is mediated by essential conserved autophagy-related genes (ATG) and is critically important for the maintenance of cell homeostasis (140, 141).

Macroautophagy, microautophagy and chaperone-mediated autophagy (CMA) are three well-recognized types of autophagy, which are characterized by various modes and different types of cargo transported to lysosomes (140). Macroautophagy (autophagy) represents the most widely studied type of autophagy. Cellular stress triggers this process, during which cytoplasmic cargo is enclosed by double-membraned autophagosomes (140). Following this, the autophagosomes fuse with lysosomes, allowing degradation of the contents and their subsequent return to the cytosol for reuse (140). Unlike macroautophagy, the lysosomal membrane invaginates or protrudes to directly engulf cytoplasmic material during microautophagy (140). However, its precise regulatory mechanisms remain largely elusive (142). CMA is characterized by transporting single cargo proteins that carry the KFERQ motif through the lysosomal membrane (140). A hallmark of autophagy is the generation of double-membraned autophagosomes, which subsequently fuse with either an endosome or a lysosome to enable degradation (143). Autophagy exhibits selectivity in degrading certain cargoes such as mitochondria (mitophagy) endoplasmic reticulum (reticulophagy), intracellular microbes (xenophagy), lipid droplets (lipophagy), peroxisomes (pexophagy) and glycogen (glyophagy) or non-selective to degrade random parts of the cytoplasm (144).

### 4.2 Autophagy of tubular epithelial cell in DKD

During the progression of DKD, defective autophagy is considered a key driving mechanism underlying tubular damage (145). Hyperglycemic conditions and the resultant metabolic disorder primarily disrupt autophagy by blocking autophagic flux and compromising lysosomal function, ultimately leading to stagnation of the autophagy pathway (146, 147). HG incubation suppresses proximal tubule cell autophagy by blocking its initiation, as characterized by decreased LC3-II levels and fewer GFP-LC3 puncta, consequently promoting cellular hypertrophy (148). Advanced glycation end products (AGEs) exposure markedly impairs autophagic flux in renal TECs by activating the SMAD3/TFEB axis to suppress lysosome biogenesis and trigger lysosome depletion, leading to the accumulation of autophagosomes and SQSTM1/p62 (41). In the mouse model of STZ-induced DKD, proximal tubule-specific knockout of *Atg5* aggravates tubule injury and kidney damage, reflected as defective autophagy sensitized proximal TECs to ferroptosis (79). Critically, autophagy is characterized by high specialization, such as mitophagy, reticulophagy and lipophagy (144). Mitophagy holds a pivotal role in the regulation of autophagy, and its dysfunction can directly cause the imbalance of mitochondrial quality control (149). In HG-induced HK-2 cells, mitoQ maintained mitophagy by restoring Nrf2 expression and activity, as well as the PINK and Parkin expression to reduce cell injury (68). Moreover, treatment with mitochondria-targeted antioxidant MitoQ preserves mitochondrial quality control, alleviates tubular injury, and restores kidney function by reversing mitophagy deficiency through the Nrf2/PTEN-induced kinase 1 (PINK) signaling pathway in db/db mice (68). Translocase of outer mitochondrial membrane 7 activation enhanced mitophagy mediated by PINK1/Parkin within TECs to attenuate kidney injury in diabetic mice (150). Optineurin suppresses mtROS-regulated NLRP3 inflammasome activation by upregulating mitophagy in HG treated TECs (151). Meanwhile, deficiency in lipophagy exacerbates lipotoxicity (85). Lipophagy defects are observed in TECs of patients with DKD and db/db mice (85). Administration of AdipoRon, an adiponectin receptor activator, mitigates tubule injury in *db/db* mice by enhancing lipophagy and suppressing ectopic lipid deposition and oxidative stress in TECs (85). Reticulophagy is known to migrate ER stress and reestablish ER homeostasis by eliminating damaged ER fragments through the autophagy-lysosome pathway. In STZ-induced diabetic mice, proximal tubule specific knockout of phosphofurin acidic cluster sorting protein 2 (PACS-2) gene severely impaired reticulophagy of tubular cells and aggravates kidney injury. *In vitro*, overexpression of PACS-2 in HK-2 cells restores reticulophagy via TFEB/family with sequence similarity 134 member B pathway (33).

Autophagy disruption is caused by multiple-level abnormalities in upstream signaling pathways (34, 117, 152). Vitamin D receptor activation reversed autophagy deficiency via AMP-activated protein kinase (AMPK)-mediated signaling in TECs, thereby ameliorating kidney injury in STZ-induced diabetic mice (34). The overactivation of ERK signaling is involved in autophagic flux disruption (117). In db/db mice and advanced glycation end-products (AGEs)-induced HK-2 cells, hyperactivation of the ERK signaling pathway contributes to the impairment of mitophagy flux via regulating TFEB in DKD (117). Lysosomal damage also participates in autophagy disruption (69, 153). In db/db mice, ameliorating lysosomal damage by melatonin restores autophagic flux in TECs, and consequently slows the development of DKD (69). Current research has shifted from a simplistic focus on enhancing autophagy to restoring autophagic homeostasis. In the discovery of therapeutic drugs, the effects of well-established autophagy regulatory agents like metformin and rapamycin are explored. AMPK agonist metformin alleviates TEC injury via enhancing autophagy in HFD/STZ-induced mice and HG-exposed HK-2 cells (154). Therapeutic strategy targeting autophagy with rapamycin obviously improves tubular damage and renal function in DKD mice (79). As a novel antidiabetic agent, glucagon-like peptide-1 (GLP-1) exerts a renoprotective effect in DKD rats via activation of autophagy through AMPK-mTOR pathway (155). The hypoglycemic drug SGLT2 inhibitors also shows promising therapeutic effect on diabetes-induced renal tubular damage by restoring autophagy (156). The effects of natural products have been also demonstrated. Melatonin, paeonol and swietenine have been found to ameliorate renal tubule injury in diabetic mice by maintaining autophagic flux of tubular cells (69, 157, 158). Targeting key signaling axes (e.g. AMPK, mTOR, and TFEB), the ultimate benefit hinges on the restoration of selective autophagic flux (34, 35, 117).

## 5 Pyroptosis

### 5.1 Pyroptotic signaling pathway

Pyroptosis features plasma membrane breakdown along with the release of pro-inflammatory cytokines (159). The execution of pyroptosis, mechanistically, relies on gasdermin protein family members (160). Mammalian gasdermins consist of a cytotoxic C-terminal domain, a flexible linker, and an N-terminal regulatory domain that is bound to and inhibited by the C-terminus (160). The pyroptotic process is typically initiated by recognition of pathogens in the cytosol or endogenous danger signals via inflammasomes, which recruit and activate inflammatory caspases such as caspase-1, -4, -5, -11 (160, 161). Once activated, these caspases cleave gasdermins to liberate the pore-forming N-terminal domains, enabling their targeting of cellular membranes to form large transmembrane pores, which disrupts cellular electrochemical gradient, and leads to water influx and cell swelling (160, 161). Ninjurin-1, a membrane protein, is also activated upon their formation (161, 162). Through oligomerization, ninjurin-1 causes the plasma membrane to rupture completely, resulting in the efflux of large damage-associated molecular patterns (161, 162).

Pyroptosis can be categorized into four well-recognized signaling cascades, namely the classical, non-classical, caspase-3-dependent, and caspase-8-dependent pathways (159, 160). The classical pathway is initiated by pathogenic stimuli, which activate pattern recognition receptors (PRRs) along with caspase recruitment domain- or pyrin domain-containing proteins (159, 163). These molecules recruit the speck-like protein, resulting in caspase-1 activation, subsequent GSDMD cleavage, and interleukins interleukin (IL)-18 and IL-1β maturation (159, 163). The activation of non-canonical pyroptotic pathways frequently occurs upon gram-negative bacterial infection (163). Upon recognizing and binding lipopolysaccharide (LPS), mouse caspase-11 or human caspase-4/5 proteolytically cleaves GSDMD, leading to plasma membrane rupture (164). The caspase-3-driven GSDME pyroptotic pathway is featured by GSDME cleavage resulting from caspase-3 activation (159). Caspase-8-dependent GSDMD pyroptosis is induced in yersiniosis via caspase-8-mediated GSDMD cleavage (163).

### 5.2 Pyroptosis of tubular epithelial cell in DKD

Emerging evidence has revealed that pyroptosis plays a significant role in DKD pathogenesis (160, 165). In DKD, TEC pyroptosis is primarily governed by the NLRP3 inflammasome signaling axis (165, 166). Under hyperglycemic conditions, SGLT2-dependent SGK1 activation drives canonical tubular epithelial cell pyroptosis, as confirmed by GSDMD-mediated pore formation and the release of IL-1β, IL-18, and LDH (48). Exosomal miR-4449 derived from DKD patient serum substantially accelerates tubular epithelial cell pyroptosis and oxidative stress through the transcriptional suppression of HIC1, leading to GSDMD-mediated pore formation and inflammatory cytokine secretion (167). Wilms tumor 1-associated protein, a core component of the m6A methyltransferase complex, promotes the modification of NLRP3 mRNA by N6-methyladenosine (166). This epigenetic regulation upregulates NLRP3 inflammasome activity, which subsequently triggers tubular cell pyroptosis, thereby exacerbating tubular damage and the progression of DKD (166). Hematopoietic prostaglandin D synthase triggers NLRP3/Caspase-1 activation to aggravate pyroptosis-mediated renal tubular injury in DKD (49). The upregulation of interferon-stimulated gene 15 in TECs disrupted mitochondrial homeostasis and triggered the release of mtDNA into the cytosol, which then triggers NLRP3‐mediated pyroptosis in cyclic GMP-AMP synthase-stimulator of interferon genes pathway (47). In db/db mice, the (pro)renin receptor triggers TECs pyroptosis through DPP4/JNK signaling mediated NLRP3 inflammasome activation (46). The inositol requiring enzyme 1 alpha (IRE1α) induced thioredoxin-interacting protein (TXNIP)/NLRP3 pathway activation and subsequent pyroptosis have been observed in both HG-exposed NRK-52E cells and diabetic rats (45). Inhibiting pyroptosis with Caspase-1 inhibitor VX-765 effectively suppressed the occurrence of pyroptosis in HK-2 cells, as well as the tubular damage, renal inflammation and DKD progression in diabetic mice (168). Collectively, these results reveal the central contribution of NLRP3 in pyroptosis. Meanwhile, non-classical pyroptosis is also involved in TECs injury in DKD. Inhibiting caspase 3/GSDME pathway signaling mediated TECs pyroptosis by dagliflozin alleviates diabetic kidney injury (169).

In the search for novel therapies for DKD, natural products have emerged as a promising source of bioactive molecules capable of inhibiting the pyroptotic pathway (170–172). Berberine, a natural alkaloid and key bioactive constituent of traditional Chinese medicinal herbs, suppresses tubular pyroptosis, thereby mitigating renal inflammation and interstitial fibrosis in DKD (170). In HFD/STZ-induced diabetic mice and HG-treated HK-2 cells, ginsenoside Rg2 prevents the TECs injury by suppressing the proinflammatory NF-κB/NLRP3 signaling pathway (171). Other natural compounds, including hyperoside, loganin, honokiol, and pyrroloquinoline quinone, have also been shown to alleviate renal damage in DKD by suppressing TEC pyroptosis (172–175). Similar therapeutic effects are observed with traditional Chinese herbal decoctions, such as the Zuogui-Jiangtang-Yishen and Tangshen formulas (176, 177). Collectively, above evidence shows that natural products may serve as effective therapeutic agents in DKD management.

Non-coding RNAs (ncRNAs), including long noncoding RNAs (lncRNAs), circular RNAs (circRNAs), and microRNAs (miRNAs), are pivotal mediators of tubular pyroptosis in DKD (178, 179). LncRNAs such as PWARSN, SNHG7, and MALAT1 have been demonstrated to drive tubular damage and the progression of DKD by activating the pyroptotic cascade (180–182). CircRNA-COL1A2 promotes pyroptosis and oxidative stress in HG-treated TECs by regulating the miR-424-5p/SGK1 pathway (183). Moreover, in DKD, circ_0004951 induces pyroptosis of renal TECs via the miR-93-5p/NLRP3 inflammasome signaling pathway (184). Meanwhile, by downregulating AIF-1/TRPC6/calcineurin A/NFAT2 axis, miR-30a regulates pyroptosis and inflammatory responses of TECs during DKD (185).

## 6 Ferroptosis

### 6.1 Ferroptotic signaling pathway

Ferroptosis is an RCD form characterized by iron dependency and lipid peroxide accumulation (186, 187). Unlike other death pathways, cells undergoing ferroptosis exhibit ruptured and blistered cell membrane, shrink mitochondria, and reduced or vanished mitochondrial cristae (186, 188). Biochemically, reactive oxygen species (ROS) generation, intracellular iron deposition, and lipid peroxides aggregation occur during ferroptosis (188–190). Despite multiple metabolic processes modulate susceptibility to ferroptosis, iron metabolism remains the central mediator of ferroptosis (189). The homeostasis of iron pool is regulated by ferritin (an iron-storage protein), iron transporters, along with transferrin and its receptor (189). When these proteins are abnormally expressed, the overload cytosolic free ferrous iron leads to intracellular ROS accumulation via the Fenton reaction, which eventually promotes lipid peroxides formation and ferroptosis occurrence (186). The iron storage protein ferritin heavy chain 1 (FTH1) enables iron store into the ferritin shell in an inert form, by converting the ferrous form (Fe2^+^) to the ferric form (Fe3^+^) (189). The xc−/glutathione peroxidase 4 (GPX4) pathway, serving as the primary antioxidant mechanism defensing ferroptosis, is another widely investigated canonical pathway (191). Comprising SLC7A11 and SLC3A2, system xc^-^ functions as a cystine/glutamate antiporter that brings extracellular cystine into cells while exporting intracellular glutamate (188, 191). As the reduced form of cystine, cysteine acts as the limiting substrate for the production of glutathione (GSH) (191). GSH is an important antioxidant against oxidative stress in cell metabolism (191). GPX4 is a crucial selenoprotein that converts toxic lipid hydroperoxides (L-OOH) on the cytoplasmic membrane to non-toxic lipid alcohol (L-OH) by utilizing GSH, thus preventing the accumulation of lipid peroxidation to protect cells from ferroptosis (191). Disruption of this axis, by either pharmacological inhibition or genetic ablation, leads to ferroptotic cell death.

### 6.2 Ferroptosis of tubular epithelial cell in DKD

Targeted interventional investigations have demonstrated that ferroptosis contributes to DKD pathogenesis (187). Under hypoxic or hyperglycemic conditions, the accumulation of HIF1α markedly drives renal tubular cell ferroptosis—characterized by HO1/TFRC upregulation, iron overload, and lipid peroxidation. AARS1-mediated lactylation of H3K18 and STAT1 substantially triggers renal tubular cell ferroptosis by transcriptionally activating the lipid-elongating enzyme ELOVL5 (56). This epigenetic modification subsequently drives PUFA biosynthesis, lipid peroxidation, and ferroptosis, as manifested by elevated LPO and Fe2^+^ levels alongside GPX4 downregulation (92). In mouse models of DKD and TGFβ-treated HK-2 cells, pharmacological inhibition of ferroptosis via the selective inhibitor Ferrostatin-1 significantly suppresses HK-2 ferroptosis, attenuates tubular injury and renal damage (81). Stabilizing ACSL4 via O-GlcNAc transferase-mediated O-GlcNAcylation facilitates TECs ferroptosis and DKD progression in HFD and STZ co-treated mice (192). Moreover, activation of the anti-oxidative defense system, such as SLC7A11 or GPX4 upregulation, confers protection against DKD by suppressing tubular ferroptosis (193, 194).

This ferroptotic process is regulated by a complex network in which transcription factors are involved (188). In db/db mice, activation of Nrf2 alleviated TECs ferroptosis, tubule damage and kidney injury (195). As a crucial regulator of lipid and glucose metabolism, AMPK serves as a critical mediator in regulating ferroptosis (196). Its inactivation increases susceptibility to tubular ferroptosis and aggravates DKD (197, 198). Consistently, AMPK activation mediates the protective effects of glucagon-like peptide-1 receptor agonists and the Tangshenning formula against TEC ferroptosis and DKD progression (197, 198). Mitochondria are essential organelles critically involved in ferroptotic cell death (186, 199). For instance, the restoration of mitophagy has been shown to suppress HG-induced tubular ferroptosis and renal damage in diabetic mice (200). The ferroptosis of TECs is also regulated by the interactive influence of the immune microenvironment (201). Deficiency of Trem2 in macrophages worsens lipid accumulation and ferroptosis in TECs via IL-1β-dependent CD36 upregulation, thus promoting DKD progression (201).

Importantly, ferroptosis has been proven to be a common intervention target for halting DKD progression (187). Whether the re-evaluation of the renal protective mechanisms of clinical hypoglycemic drugs (SGLT2 inhibitors and GLP-1 receptor agonists), the multi-target advantages of traditional Chinese medicine products (such as naringenin, astragaloside IV), or the application of nanomaterials, all these interventions delay the progression of DKD by blocking tubular cell ferroptosis (202–207). Collectively, these studies, ranging from molecular mechanisms to translational applications, systematically establish ferroptosis as a central link connecting metabolic disorders, oxidative stress, and structural damage to renal tubule, thereby providing a new theoretical basis and intervention targets for DKD diagnosis and therapy.

## 7 Emerging modalities of RCD in diabetic tubular injury

Recent research has linked the pathogenesis of tubular injury to other new forms of RCD pathways, such as PANoptosis, cuproptosis, and NETosis, Parthanatos and lysosome-dependent cell death.

### 7.1 PANoptosis

PANoptosis is a newly recognized, highly coordinated inflammatory cell death pathway that integrates key molecular components of pyroptosis, apoptosis, and necroptosis within a single macromolecular complex called the PANoptosome (208). PANoptosis-related gene signatures are strongly enriched in the renal tubulointerstitial tissue of patients with DKD, serving as a comprehensive indicator of tubulointerstitial injury (209–211). Several hub genes of PANoptosis, such as CASP1, YWHAH, and TAP1, are significantly upregulated during DKD progression, correlating with glomerular filtration rate decline and clinical severity (209, 212). HG condition significantly increased TAP1 and CASP1 expression levels in HK-2 cells (212). Therapeutic agents like finerenone can effectively modulate the PANoptotic cascade to alleviate local immune infiltration and tubular damage (213).

### 7.2 Cuproptosis

Cuproptosis is an oligoylated, copper-dependent form of cell death triggered by the direct binding of excess intracellular copper to lipoylated proteins involved in the tricarboxylic acid (TCA) cycle (214). Because renal TECs are highly metabolically active and heavily rely on mitochondrial oxidative phosphorylation, they are vulnerable to cuproptosis-mediated damage (215). FDX1 and LIAS, two key regulators of cuproptosis, are consistently upregulated in bulk RNA-seq, single-cell RNA sequencing, and spatial transcriptomics analyses in human samples (216). The expression of FDX1 and LIAS proteins were also significantly increased in the renal tissues in db/db mice, compared to db/m controls (216). Cuproptosis-related gene signatures, including ITGB6, LTBP1, FSTL1, CX3CR1, and AGR2, are significantly upregulated in DKD, serving as robust diagnostic biomarkers that correlate with immune cell infiltration and progressive tubulointerstitial damage (217).

### 7.3 NETosis-related tubular injury

NETosis is a specialized form of neutrophil cell death where neutrophils extrude their nuclear chromatin and granular proteins to form neutrophil extracellular traps (NETs) (218). Release of highly cytotoxic components of NETs can directly damage adjacent renal TECs, exacerbating endothelial dysfunction, podocyte damage, and fibrotic remodeling (219). Upregulated humoral factors, such as Midkine, further promote tubulointerstitial injury by accelerating local NETosis and subsequent tubular cell death (220). In silico profiling has demonstrated that the severity of diabetic tubulointerstitial injury correlates significantly with the dysregulation of CASP1 and LYZ, both of which represent pivotal hub genes orchestrating NET formation and clearance (221).

### 7.4 Parthanatos and lysosome-dependent cell death

Parthanatos is a form of RCD initiated by the hyperactivation of poly (ADP-ribose) polymerase 1 (PARP1) in response to massive oxidative stress and DNA damage (222). Lysosome-dependent cell death (LDCD), on the other hand, is mediated by lysosomal membrane permeabilization (LMP) under metabolic stress (223). The activity level of parthanatos was decreased, while the activity level of LDCD was increased in DKD compared with control, as shown by a bioinformatic study (135). The diagnostic efficacy of LDCD pathway activity was 0.7001 (135).

## 8 Interaction between RCD of TECs in DKD

In the pathology of DKD, renal tubular cell fate is tightly regulated by multiple modes of RCD (224). Substantial evidence has demonstrated that apoptosis, pyroptosis, ferroptosis, and autophagy form an intricate regulatory network, collectively determining the progression of tubular injury through shared signaling pathways and phenotype transition (200, 225, 226) (**Figure 2**). Among these RCD modalities, autophagy shows dual effect on tubular damage under different conditions (200). In diabetic mice, rescuing PINK1-mediated mitophagy by caffeic acid phenethyl ester could protect TECs against ferroptosis (79, 200). PINK also exerts protective effects on diabetic tubular injury by suppressing necroptosis via inhibiting mtROS (30). Conversely, autophagic deficiency acts as a triggering factor for other death pathways (79). Defective autophagy heightens TECs susceptibility to ferroptosis in DKD, demonstrating the close correlation between autophagy flux impairment and ferroptosis (79). The reticulophagy restoration mediated by glutathione s-transferase kappa 1-reticulophagy regulator 1 ameliorated DKD by suppressing tubular apoptosis (37).

Regarding the transition between different forms of RCD, a definitive molecular switch exists between apoptosis and pyroptosis, connecting non-inflammatory and inflammatory cell death (227). Study has demonstrated that GSDMD mediates the shift of tubular cells from apoptosis to pyroptosis in TLR4-regulated diabetic tubular damage, suggesting that GSDMD activation promotes the conversion from non-inflammatory apoptosis to inflammatory pyroptosis (227). Despite being mechanistically different, pyroptosis, ferroptosis, apoptosis, necroptosis act synergistically via shared signaling nodes in DKD, with mitochondrial ROS (mtROS) and serving as a pivotal common mediator (30, 112, 176, 207). Zuogui-Jiangtang-decoction represses renal TECs pyroptosis through blocking mtROS -NLRP3 signaling in DKD (176). Similarly, adaptive antioxidant nanomedicines suppress mtROS to inhibit TECs ferroptosis to alleviate DKD (207). Nrf2 serves also as a common regulator in modulating autophagy, pyroptosis and ferroptosis. MitoQ alleviates tubular injury and restores kidney function by reversing mitophagy deficiency through the Nrf2/PINK signaling pathway in DKD mice (68). In db/db mice, activation of Nrf2 alleviated TECs ferroptosis, tubule damage and kidney injury (195). Syringaresinol also protects against diabetic tubular by suppressing TECs pyroptosis via Nrf2-mediated antioxidant pathway (52). AMPK regulates autophagy and ferroptosis, as well as acting as a negative modulator of mtROS (34, 79, 198). Furthermore, Bim, the downstream mediator of JNK, shows dual effects on autophagy and apoptosis (116, 228). Beyond these, in the tubular injury of DKD, autophagy determines cellular susceptibility to apoptosis, pyroptosis and ferroptosis (37, 79, 225). These interactions suggest that targeting a single mode of cell death may be insufficient to fully attenuate tubular injury. Therefore, multi-target intervention strategies may provide promising therapeutic approach for managing DKD.

## 9 Clinical relevance of RCD pathways

Recent clinical data mining utilizing specific RCD-related gene signatures has successfully recapitulated the multifaceted manifestations of RCD in human DKD biopsy samples, highlighting its central role in driving disease progression and immune microenvironment remodeling. The core genes and diagnostic value of analyzed RCD are summarized in **Table 3**. In integrated transcriptomic and single-nucleus RNA sequencing (snRNA-seq) analyses, necroptosis was identified as a highly enriched RCD pattern in DKD tubules, defining distinct patient subtypes that correlate closely with chronic inflammatory responses and macrophage infiltration (229). Similarly, bioinformatic models using eight key ferroptosis-related gene signatures, or a six-biomarker diagnostic signature centered on TP53, have demonstrated exceptionally high diagnostic accuracy in distinguishing DKD from controls (231, 232). In parallel, integrated analyses of pyroptosis-related differentially expressed genes have identified a ten-hub-gene signature that is intimately linked with the upregulation of pro-inflammatory M1 macrophage infiltration in human DKD renal tissues (233). To resolve the complex crosstalk among these pathways, machine-learning frameworks built on PANoptosis-related risk scores (PRS) comprised of six key hub genes (YWHAH, PRKACB, PSMB9, FAS, GZMA, and CASP1) have revealed extensive molecular co-activation within tubular and interstitial segments, strongly predicting eGFR decline, serum creatinine elevation, and local immune cell recruitment (209). Crucially, an integrated, snRNA-seq-based comparison across 13 distinct PCD modalities established entotic cell death, apoptosis, necroptosis, and pyroptosis as the four dominant cell death pathways driving DKD progression (135). This study further developed a cell death-related signature (CDS) based on CASP1, CYBB, PLA2G4A, and CTSS, providing an effective diagnostic tool that tightly correlates with progressive renal function decline and local immune microenvironment remodeling (135).

**Table 3:** Clinical diagnostic value of different RCD pathways.

RCD	Dataset	Core genes	Diagnostic value (AUC)
Necroptosis	GSE131882, GSE96804, GSE142025	EGF, PAG1, ZFP36	0.877 – 0.974 (229)
Necroptosis	GSE96804, GSE30122, GSE30528	CFLAR, FMR1, GSDMD, IKBKB, MAP3K7, NFKBIA, PTGES3, SFTPA1	0.81 - 0.96 (230)
Ferroptosis	GSE96804, GSE30566, GSE99339, GSE30528	SKIL, RASA1, YTHDC2, SON, MRPL11, HSD17B14, DUSP1, FOS	0.818 (231)
Ferroptosis	GSE30122, GSE1009	TP53, RB1, NF2, RRM2, PRDX1, CDC25A	0.863 - 0.993 (232)
Pyroptosis	GSE96804, GSE131882	ECM1, LRP2BP, ALKBH7, CDH10, DUSP1, HSPA1A, LPL, NFIL3, PDK4, and TMEM150C	0.883 - 0.999 (233)
PANoptosis	GSE104954, GSE30122, GSE47185, GSE99325	YWHAH, PRKACB, PSMB9, FAS, GZMA, and CASP1	0.981 (209)
Multiple RCDs	GSE30528, GSE47183, GSE96804, GSE99339, GSE104948, GSE131882	CASP1, CYBB, PLA2G4A, CTSS	0.756 – 0.883 (135)

RCD, Regulated Cell Death; AUC, Area Under the Curve; EGF, Epidermal Growth Factor; PAG1, Phosphoprotein Associated With Glycosphingolipid Microdomains 1; ZFP36, ZFP36 Ring Finger Protein; CFLAR, CASP8 And FADD Like Apoptosis Regulator; FMR1, Fragile X Messenger Ribonucleoprotein 1; GSDMD, Gasdermin D; IKBKB, Inhibitor of Nuclear Factor Kappa B Kinase Subunit Beta; MAP3K7, Mitogen-Activated Protein Kinase Kinase Kinase 7; NFKBIA, NFKB Inhibitor Alpha; PTGES3, Prostaglandin E Synthase 3; SFTPA1, Surfactant Protein A1; SKIL, SKI Like Proto-Oncogene; RASA1, RAS P21 Protein Activator 1; YTHDC2, YTH Domain Containing 2; SON, SON DNA and RNA Binding Protein; MRPL11, Mitochondrial Ribosomal Protein L11; HSD17B14, Hydroxysteroid 17-Beta Dehydrogenase 14; DUSP1, Dual Specificity Phosphatase 1; FOS, FOS Proto-Oncogene, AP-1 Transcription Factor Subunit; TP53, Tumor Protein P53; RB1, RB Transcriptional Corepressor 1; NF2, NF2, moesin-ezrin-radixin like (MERLIN) tumor suppressor; RRM2, Ribonucleotide Reductase Regulatory Subunit M2; PRDX1, Peroxiredoxin 1; CDC25A, Cell Division Cycle 25A; ECM1, Extracellular Matrix Protein 1; LRP2BP, LRP2 Binding Protein; ALKBH7, AlkB Homolog 7; CDH10, Cadherin 10; HSPA1A, Heat Shock Protein Family A (Hsp70) Member 1A; LPL, Lipoprotein Lipase; NFIL3, Nuclear Factor, Interleukin 3 Regulated; PDK4, Pyruvate Dehydrogenase Kinase 4; TMEM150C, Transmembrane Protein 150C; YWHAH, Tyrosine 3-Monooxygenase/Tryptophan 5-Monooxygenase Activation Protein Eta; PRKACB, Protein Kinase cAMP-Activated Catalytic Subunit Beta; PSMB9, Proteasome 20S Subunit Beta 9; FAS, Fas Cell Surface Death Receptor; GZMA, Granzyme A; CASP1, Caspase 1; CYBB, Cytochrome B-245 Beta Chain; PLA2G4A, Phospholipase A2 Group IVA; CTSS, Cathepsin S.

## 10 Conclusion and perspectives

DKD, a serious DM-related microvascular disorder, is pathologically defined by persistent hyperglycemia-triggered complex metabolic abnormalities, ultimately leading to glomerular filtration barrier destruction and tubulointerstitial injury (8, 10). RCD plays a central role in DKD, overactivated TECs apoptosis causes tubular injury, autophagy deficiency disrupts cellular energy recycling and homeostasis, ferroptosis exacerbates mitochondrial injury via lipid peroxidation and iron overloading, and pyroptosis causes overactivated local cascadic inflammatory response to aggravates renal damage (13, 132, 224). These different types of RCD collectively form complex pathological networks underlying diabetic kidney injury.

Traditional research primarily focused on individual RCD pathways, based on the hypothesis that each form of RCD occurred in isolation (129, 169, 198, 206). However, emerging evidence has revealed the interaction among different types of RCD (226, 227, 234). GSDMD acts as a pivotal regulatory protein that simultaneously controls apoptosis and pyroptosis, thereby obscuring the boundaries between these processes (227). Autophagy regulates ferroptosis through mechanisms such as mitophagy and ferritinophagy (200, 235). Collaborative effects also exist between ferroptosis and pyroptosis (176, 207). The complex regulatory interactions among RCD suggest that the current strategies targeting a single death pathway possess inherent limitations.

At present, numerous interventional strategies have demonstrated nephroprotective effects by targeting tubular cell death in DKD (173, 176, 200, 204–206). The therapeutic mechanisms of established clinical drugs are being re-evaluated. SGLT2 inhibitors exert multiple effects, including activating autophagy and suppressing pyroptosis and ferroptosis, to mitigate tubular injury and kidney damage (119, 156, 205, 206). Traditional Chinese medicine and natural products possess unique advantages in simultaneously intervention on multiple modes of RCD (131, 176, 177, 194, 202). Collectively, these findings suggest that the future therapeutic strategy may shift from single pathway blockage to multi-target homeostasis restoration, thereby enhancing clinical efficacy.

The onset and the interactions of tubular cell RCD in the pathogenesis of DKD remain to be further investigated. Future studies should focus on spatiotemporal dynamics, the dominant roles of different forms of RCD, and the identification of the key molecular switches that govern their interactions. Furthermore, a critical remaining gap in current translational research is the lack of stage-specific and pathway-specific efficacy data for established therapeutic agents. Although SGLT2 inhibitors, metformin, and other agents are well-documented to modulate multiple RCD pathways, it remains unclear whether their clinical renoprotective efficacy is strictly dependent on the specific cell death modality dominant at different stages of DKD progression. Future investigations employing sequential single-cell transcriptomics, specific inducible gene knockouts, and clinical cohort stratification are urgently needed to elucidate these temporal dependencies, which will pave the way for precise, stage-targeted RCD-modulating therapies in DKD. Moreover, developing molecules or chimeric drugs capable of simultaneously blocking multiple cell death pathways may be promising DKD therapeutic approaches, potentially providing patients with more precise and effective treatment options.
